# Current Therapeutic Strategies in Treating Obesity in Children and Adolescents – Review of The Literature

**DOI:** 10.34763/devperiodmed.20172103.286292

**Published:** 2017-10-28

**Authors:** Agnieszka Zachurzok, Anna Springwald, Piotr Gibała, Paweł Matusik

**Affiliations:** 1 School of Medicine in Katowice, Department of Pediatrics and Pediatric Endocrinology, Medical University of Silesia in Katowice, Katowice Poland

**Keywords:** obesity, children, behavioral treatment, physical activity, otyłość, dzieci, terapia behawioralna, aktywność fizyczna

## Abstract

The present paper is a review of current knowledge concerning the most appropriate strategy in the conservative therapy of obese children and adolescents. It presents an account of the combined mode of therapy consisting of behavioral and dietary counseling with appropriate physical activity (also using the support of modern media and devices). The rise in obesity is usually a long-lasting process involving genetic predispositions but also environmental factors which can be modified. The treatment of obesity should be focused on these modified factors and be a lifelong treatment, not merely a short period of exercise or a diet program. Successful therapy depends on the cooperation of the medical team with children and their parents or caregivers.

## Streszczenie

W pracy przedstawiono aktualną wiedzę na temat najbardziej skutecznych strategii w zachowawczym leczeniu otyłych dzieci i młodzieży. Szczegółowo omówione zostało leczenie kombinowane będące połączeniem postępowania behawioralnego, dietoterapii z odpowiednią aktywnością fizyczną (przy wykorzystaniu nowoczesnych mediów i urządzeń). Rozwój otyłości jest zwykle procesem długotrwałym, obejmującym predyspozycje genetyczne, ale także modyfikowalne czynniki środowiskowe. Leczenie otyłości nie może być tylko krótkim okresem ćwiczeń lub programem dietetycznym. Powinno się ono koncentrować właśnie na czynnikach modyfikowalnych, a dokonana zmiana musi być utrzymana przez całe życie. Powodzenie terapii jest uzależnione od współpracy zespołu medycznego z dziećmi i ich rodzicami lub opiekunami.

## Introduction

In recent decades overweight and obesity among children and adolescents has become a serious public health problem. According to the data for 2011 published by the International Association for the Study of Obesity, 12.4% Polish girls and 16.3% Polish boys were overweight or obese [1]. Prospective studies showed that excessive weight at the age of 15-17 years is associated with a 17.5-fold risk of obesity in adulthood. It is assessed that 80% of obese adolescents become obese adults, copying unhealthy dietary habits from their families [2]. Moreover, excessive weight in childhood is associated with its metabolic consequences: carbohydrate and lipid disturbances, insulin resistance and in the youngsters’ future life - type 2 diabetes, as well as cardiovascular diseases [3]. The rise of obesity is usually a long-lasting process involving genetic predispositions but also environmental factors which can be modified [4]. The treatment of obesity should be focused on these modified factors and be a lifelong treatment, not merely a short period of exercise, or a diet program.

## Team work

The main aspect of childhood obesity treatment is a multidisciplinary intervention approach involving:

1. Diet modification – both qualitative as well as quantitative,
2. Lifestyle modification – physical activity versus a sedentary lifestyle,
3. Behavioral changes

A professional team, consisting of a physician, dietician, physiotherapist and psychologist should be involved in the treatment of obese children, especially in subjects with already present metabolic disturbances or at high risk of contracting them. The main target of the therapeutic team should be to provide assistance to the child and family and help them implement gradual changes in their diet and lifestyle in order to abolish the imbalance between calorie intake and expenditure [5, 6, 7]. Children and their families should be incorporated into the therapeutic process as well. The target of the treatment is to teach them how to make healthy food choices, increase physical activity and implement desirable changes. The intervention should be tailored to the age of the patient, the severity of obesity and the present complications, but should also be adapted to the needs of individuals, so as to be accepted by both the subjects and their families. A well-balanced, diverse diet should be promoted, and particular attention should be given to regularly eating breakfast, consuming an appropriate amount of vegetables and fruits and limiting sweet and high-calorie beverages and soft drinks [8]. Restrictive diets, very-low-calorie ones, as well as diets with special, processed diet food are contraindicated in children. What is recommended is to increase physical activity and decrease the time spent in a sitting and lying position. Moreover, it is important to take into consideration the psychological and social aspects of the patients and their family life. Therefore, it should be considered whether psychotherapeutic consultation is required [9].

## Behavioral changes – From diagnosis to counseling

The first step in the therapeutic approach is the assessment of lifestyle and diet. Detailed questions about the amount and quality of the food consumed, as well as the estimation of the time spent on physical activity and sedentary behaviors (watching TV, playing on the computer) could show the reason for being obese (e.g. excessive juice consumption, skipping breakfast, overeating in the evenings) and be the starting point for the construction of a therapeutic plan. What can be very helpful is a diet and physical activity diary kept for a couple of days or one week by the patient or a parent. On the basis of the information that is gathered an assessment of the patient’s and his/ her family habits could be performed [8,10]:

1. The number of meals eaten per day, their caloric content and nutritive value,
2. The amount and caloric content of beverages, soft drinks and juices,
3. How often meals are eaten out (at bars, restaurants, fast food bars)
4. How often, where, and with whom the meals are eaten at home, who is responsible for preparing meals, what is the patient’s/ the family’s favourite food, etc.,
5. The socioeconomic status of the family,
6. Previous efforts to change the diet and lifestyle

In most cases it is possible to identify one or a couple of problems involved in obesity (the size of the meal, beverages between meals, soft drinks, irregular eating) on the basis of such an evaluation. However, sometimes identifying the causes is not enough, and a psychologist’s input is needed to find the reason for the unhealthy eating behaviors (e.g. compulsive eating):

1. Emotional, psychological problems,
2. Family or environmental functioning problems,
3. Seeking pleasure and acceptance in unhealthy food,
4. The cultural or social context of the family (feasting),
5. Environmental factors (advertisements of unhealthy food and unhealthy food accessibility) [10].

The next step is to define the goal – the reduction of body weight and achievement of proper body proportions. In the overweight child a reduction of the BMI is achieved by:

1. An increase of 1 kg of body weight for every 2 cm of body height in children between 2 - 4 years old,
2. Maintaining constant body weight leading to a decrease of the BMI with increasing height in children over the age of 4.
  If the child is obese, the goal of the treatment is:

1. Maintaining constant body weight leading to decreasing the BMI with increasing height in younger children,
2. Reduction of body weight by 1-2 kg per month in teenagers [9].

The main target, i.e. BMI reduction, should be determined by small, step-by-step goals, which are realistic and accessible for both the patient and the family. Behavioral therapy is based on the concept that obesity is a “learned disease” which can be treated by “re-learning”. The therapy is based on the concept of bad eating habits in which the insufficient control of stimulus-or-reward behavior results in increased food intake. These habits can be broken down into small sequences and rebuilt into desirable ones. The target of the therapy is to make small changes in the diet and behavior of the child and the family by:

1. Learning to make healthy food choices,
2. Learning to feel satiety and hunger,
3. Learning how to eat regularly and slowly,
4. Increasing physical activity,
5. Decreasing sedentary behaviors (watching TV, playing on the computer) [9].

The re-learning process concerns not only the child but also the whole family. The caretakers should, therefore, learn how to:

1. Not use food as a reward,
2. Say “no” to a child asking for food,
3. Not stigmatize your child, not make him/her feel isolated – the whole family should change their diet and eat the same food,
4. Do the shopping together with the child, check the amount of fat, sugar, calories in products,
5. Prepare meals together with the child,
6. Eat at home, at the table, together,
7. Not allow eating while watching TV and playing on the computer,
8. Limit the availability of sugar-sweetened beverages, soft drinks, sweets,
9. Allow the child to make his/ her own decisions - give two suggestions of meals to choose from,
10. Choose the time and quality of the meal but the child decides how much food he/ she eats,
11. Not allow to skip meals, especially breakfast,
12. React to unhealthy food advertisements,
13. Make changes in the life of the whole family – “do what I do” not “do it, because I say so”,
14. The whole family “speaks with one voice” – the therapeutic team also involves grandparents, aunts and uncles [11].

## Diet modification

The amount and quality of food in a child’s and adolescent’s diet is crucial to supply the adequate number of calories and nourishing components necessary for normal growth and development. Diet modification should lead to a decrease of calories and improvement in the balance of the food consumed, without excessive restrictions in the amount of food [12]. It should be based on 4-5 meals a day with 60-65% low glycaemic index carbohydrates, 10-12% proteins and 25% fat [12]. The energy intake should be adequate for the age of the children and for their energy expenditure. It is worth remembering that more physically active children and adolescents will require additional calories. The elimination of any group of food, even sweets, from a child’s diet is contraindicated. About ¼-^1^/_3_ of all the calories eaten by teenagers come from sugar-sweetened beverages and soft drinks, so in diet modification we should focus on decreasing or abolishing the consumption calorie-dense, low nourishing food, especially [12]:

1. High calorie, sugar-sweetened or salty beverages,
2. Soft drinks,
3. Fruit juices and juicy drinks,
4. Fast food and vending machine food

soft drinks and fruit juices should be replaced by water. According to the American Academy of Pediatrics, a child up to the age of 6 years can consume 125 ml of natural, 100% fruit juice per day, an older child – 200 ml per day [13]. It should be remembered that juice is a source of calories but not of satiety. It is recommended that small amounts of sweets be served 3 - 4 times per week in a controlled way.

A complete elimination of sweets from a child’s diet could lead to their uncontrolled and excessive consumption. Low-fat sweets are indicated – gummy-bears, candies, jellies or sorbets. It is worth trying to add healthy snacks (fruits) with a low-calorie content (vegetables) to everyday diet [14, 15].

It is recommended for every child to consume [13]:

1. Fruit and vegetables – 5 servings per day,
2. Dairy products – 3 servings per day,
3. Complex carbohydrates (whole-grain products) – with every meal,
4. Meat, fish, eggs – 1 – 2 servings per day,
5. Fat – in a limited amount, mainly of plant origin,
6. Sweets – in a limited amount, without the trans fat content,
7. Salt – in a limited amount,
8. Water – in an unlimited amount, during and between meals [13].

The target is the consumption of 3-4 servings of fruit or vegetables per day for younger children and 4-5 servings for older ones. One serving of fruit means one average-size fruit, ¼ cup of dried fruit, ½ cup of fresh or frozen small fruit or ½ cup of juice. Fruit is a source of sugar (fructose, saccharose), so it should not be eaten between meals in an unlimited amount. It is recommended that fruit should be incorporated into meals: first-, second-breakfast, dessert. High-sugar fruit (grapes, bananas) should be avoided [13]. One serving of vegetables means 1 cup of a raw leafy vegetable, ½ cup of a cut-up raw or cooked vegetable, ½ cup of vegetable juice. Many raw vegetables contain so few calories and their glycemic index is so low that they can be consumed as a healthy beverage between meals [13]. Diets rich in fruit and vegetables usually contain a high amount of potassium, antioxidants and trace elements leading to a decrease of the cardiovascular risk.

An adequate intake of dairy products is recommended. The reduction of overweight up to 70% can occur by eating at least two dairy servings per day. Moreover, appropriate calcium intake, essential for proper growth, can also reduce the risk of insulin resistance and type 2 diabetes. Whole-fat milk and dairy products are the main source of saturated fat and cholesterol in a child’s diet. It is recommended that saturated fat consumption should be reduced to below 10% of the daily caloric demand in children over the age of 1 year. In children between the age 1 and 2 years 2% fat milk is suggested in order to avoid a deficiency of fat-soluble vitamins and fatty acids essential for central nervous system myelination. For children older than 2 years low-fat milk and dairy products are suggested [12, 13]. The consumption of butter, cream, hard and processed cheese (because of its high saturated fat content), as well as sugar-sweetened cottage cheese and yogurts should be reduced. Low-fat milk, cottage cheese and yogurt are recommended [13].

High dietary fat intake may displace more micronutrient-dense, fibrous food, leading to the deterioration of nutrient adequacy and diet quality. Fat-rich pork meat and processed pork as well as gravies (cheese, mayonnaise) should be eliminated. It is recommended that the consumption of poultry (without skin), fish, nuts and plant oils should be increased. Meat should be broiled, roasted, grilled or poached, not fried. Fish should be eaten at least twice a week.

Carbohydrates of proper quality should be incorporated in each meal. Complex carbohydrates with a low glycemic index (brown rice, whole-wheat bread) are more suitable than easily absorbed, high-glycemic ones (white bread, white rice, white <our products). Besides a lower glycemic index, they also contain more fiber. High fiber consumption from fruit, vegetables and wholemeal products increases the feeling of satiety, postpones the feeling of hunger, decreases LDL-cholesterol concentration and insulin resistance. Moreover, it leads to the reduction of energy-dense food consumption due to changes in the type of food proportions.

## Number, size and time schedule of meals

The number, time schedule and the size of meals should be adapted to the age of the child. It is recommended that 3 bigger and 2 smaller meals per day should be eaten. Breakfast is a very important meal – its consumption reduces snacking during the day [12]. The evening meal should be hearty and eaten at least 2 hours before going to bed. Snacking of sugar-sweetened or salty beverages between meals is contraindicated.

The number of calories consumed is related to the size of the meal. However, if the size of the meal is significantly decreased, there will be no feeling of satiety. It is, therefore, recommended that the size of the meal should be decreased very gently while significantly cutting the numbers of calories. A simple strategy to lower the energy density of the meal involves reducing fat and adding water-rich foods, such as soups, vegetables, and fruit. Meals should be served on small plates and second helpings should be avoided. A well-balanced meal should contain ½ a plate of vegetables, ¼ of a plate of meat, fish or eggs and ¼ of a plate of potatoes, whole-wheat pasta, bread or brown rice. It is extremely important to teach the child to recognize satiety and hunger. Moreover, the surroundings: i.e. the place and time of the meal are very important. The meal should be eaten:

1. at home, at the table,
2. with family members – starting the meal together and finishing together,
3. at a fixed time of the day,
4. unhurriedly,
5. not while watching TV or playing on the computer [12-14].

## Useful diets

For a long-term weight control program, lifestyle-modification diets are usually most successful (e.g. the Mediterranean diet, the traffic-light diet). The Mediterranean diet promotes the consumption of fresh fruit, vegetables, whole grain products, and olive oil. It is very effective in reducing the risk of cardiovascular diseases and is a well-balanced diet, containing all groups of products [16]. The traffic-light diet divided foods into three colour-coded groups. Red indicates high-fat, high-calorie and nutrient-poor foods that should not be eaten or can be eaten rarely (a maximum of 4 times per week); yellow – moderate-calorie foods that can be consumed in limited amounts; while green refers to vegetables and some fruits which can be eaten freely [17]. The traffic-light diet is associated with an improvement in nutrient density for protein, calcium, iron, vitamin A, thiamine, riboflavin and a decrease in nutrient density for fat [18].

## Physical activity

Nowadays another very important cause of the global increase in childhood obesity is the markedly lower level of physical activity (PA) among children and adolescents. Active behaviors have been displaced by more sedentary pursuits, which have contributed to a reduction in physical activity involving energy expenditure. These days children are at a high risk of living life less healthily than their parents [19].

It is not surprising that obese children have impaired physical functions compared to their healthy-weight peers. It was documented that children with a higher body fat percentage have poorer knee extensor strength, cardiorespiratory fitness, and overall physical functioning [20]. Moreover, decreased locomotor capacity, physical health-related quality of life (HRQOL) and reduced time spent in community participation activities were confirmed, as anticipated [21, 22].

## Benefits from PA

Regular PA improves motor skills, musculoskeletal structures, metabolic and cardiovascular functions. It leads to numerous adaptive responses of the organism. Physical training results in more efficient oxygen transfer to muscles, therefore it is able to better utilize the unlimited lipid stores instead of the limited reserves of carbohydrates [23].

Studies highlight the essential role of physical activity in child health and development. There is extensive available evidence that regular physical activity prevents pediatric overweight and obesity [24]. Public health recommendations are aimed at promoting and maintaining general health in children and adolescents and it must be emphasized that they are not aimed at losing weight and treating pediatric obesity. We have to notice that “threshold”. When it comes to treating childhood obesity and its related comorbidities, and not only preventing it, moving beyond that threshold can be necessary. This requires adapted techniques and therapeutic exercise strategies [25].

## Basic recommendation

Before considering any specific exercise intervention, general practitioners and pediatricians should encourage daily active play and movement and in this way promote reducing sedentary behaviors. This is the first and natural step for children. It is important for them to keep this in mind throughout their life. WHO guidelines recommend that 5-18-year-old children should allow a minimum of 60 minutes per day for PA and children under 6 years old at least 3 hours per day while playing and exercising. On the other hand, the maximum screen time for children over 5 years old is 2 hours per day, and for those 2-5 years old not more than 1 hour a day. Current studies show that few children and adolescents meet these recommendations [26, 27].

As far as severely obese children are concerned, it should be taken into consideration that the exercise tolerance in these children is very low. In such cases special supervision during a specifically graded program is required [28].

## Effects of physical activity

Physical activity should be accompanied by classical treatment of obese children. This leads to achieving better results of the treatment. Even short periods of cycling, walking or aerobic training on a daily basis improve the results of obesity treatment. [29, 30]

There are several methods of physical activity which are used as treatment in the population of obese children. These methods are combined with behavioral intervention, diet and modification of lifestyle. Multi-component behavioral intervention includes physical activity and change of behavior. Diet and modification of lifestyle may be beneficial in achieving weight reduction [31].

## Family-Based treatment (FBT)

FVT is a behavioral weight control intervention program developed especially for children who are overweight and obese and for their parents. It uses physical activities and is provided to improve the weight status of the patients. FBT has significant positive effects on the BMI in children. According to Boutelle et al. the loss of the BMI of the mean child involved in the study was 0.25 kg/m^2^ after 6 months of intervention. This model of treatment leads to weight loss of the parent and child and is a gold-standard treatment for childhood obesity [32]. Moreover, Ranucci et al. noticed that both children and adolescents showed a significant reduction in body weight, BMI and waist-to-height-ratio after the intervention. The family-based treatment was also effective in improving the health status, nutrition habits, and physical performance in children and adolescents [33].

Exercise training improves cardiovascular fitness and muscle strength. It has an influence on the blood lipid profile and blood pressure in obese children. In their study Czyż et al. showed that physical fitness was statistically significantly lower in the group of obese children they investigated compared to normal weight and even underweight children [34]. Intervention programs that included physical fitness through aerobic training played an important role in reducing the obesity in children [34]. Moreover, exercise training is associated with the reduction of fasting insulin levels and insulin resistance (measured by HOMA-IR) in children with obesity. It may prevent type 2 diabetes and other metabolic syndrome components in obese children and adolescents [35].

## Web-Based interventions

Using web-based technology devices has become a normal part of children’s and adolescents’ life nowadays. Taking advantage of this phenomenon could have a significant impact on increasing knowledge about healthy lifestyle. Using it as an educational tool may be an effective method to provide more personalized intervention to decrease the BMI z-score in obese school-age children.

Mohammed Nawi et al. conducted a trial in which one group of adolescents was provided with information on healthy lifestyle and diet via the Internet (obeseGO!). The control group were provided only with pamphlets on health education. The 3-months’ trial showed a small effect in controlling and reducing overweight and obesity online compared to health education via a pamphlet [36]. In the study of Sousa et al. the intervention group of children from a pediatric obesity clinic was invited to access an e-therapeutic platform (Next.Step) over 24 weeks in addition to the standard treatment program. The control group was provided only with the standard treatment program. While comparing the BMI and percentage of fat mass in both groups, the outcomes were not significantly better in the intervention group. Later a significant increase in health responsibility after the e-therapeutic program was observed [37]. Based on the above studies we can conclude that other well-designed web-based intervention programs are needed.

## Mobile apps and games

Mobile apps and games with or without sensors were the most commonly employed technology in pilot intervention and user studies and often focused on accurately measuring or promoting increased PA behaviors [38, 39, 40].

Among the most popular ones is the app of Pokémon Go, which was confirmed to have led to significant increases in physical activity over a period of 30 days with particularly engaged users [41].

## Sms intervention

De Niet et al. observed in their study an intervention group who for 9 months received a short message service maintenance treatment (SMSMT) and compared it with a control group. The intervention group sent self-monitoring data on exercise every week via mobile phones. In return they received tailored feedback messages. No positive effect of SMSMT on weight was found in the intervention group compared to the control group of children [42]. Moreover, other studies confirmed that the reduction of weight in obese children due to SMSMT has no statistical significance [43, 44].

## Use of pedometer

This is another method of encouraging children and adolescents to increase their PA. It involves cheap and easy-to-handle devices. Staiano et al. in their study divided obese children into three cohorts: no –pedometer, pedometer only and pedometer with step goals. The trail showed that over 10 weeks the children provided with pedometers with goals significantly reduced both their BMI and BMI z-scores compared with the no-pedometer group. They also achieved a significant increase in steps-per-day compared with the pedometer-only group [45]. By contrast, the study of Currie et al. showed that among severely obese children the change in their BMI z-scores was similar among those who did and did not successfully increase PA levels with a pedometer [46].
